# Diagnostic Approach for Stroke Etiology in Takayasu Arteritis

**DOI:** 10.7759/cureus.8394

**Published:** 2020-06-01

**Authors:** Furkan M Yilmaz, Hamza Shaikh, Ryna Then

**Affiliations:** 1 Neurocritical Care, The Ohio State University Wexner Medical Center, Columbus, USA; 2 Neurosurgery and Radiology, Cooper University Hospital, Camden, USA; 3 Neurology/Vascular Neurology, Cooper University Hospital, Camden, USA

**Keywords:** takayasu disease, t1 fat suppression, stroke, arteritis, magnetic resonance imaging

## Abstract

Takayasu arteritis (TA) is a rare inflammatory arteritis that is usually affecting young women and causes ischemic changes in the vessel wall. In this report of TA leading to an acute ischemic stroke, we describe a treatment-resistant case, with short interval flares and the challenge of defining the stroke etiology as a large vessel occlusive disease vs. an arteritis flare. We have used CT and MRI modalities to show the active disease and got diagnostic answers from this tool.

## Introduction

Takayasu arteritis (TA) is a rare large vessel inflammatory arteritis that is usually affecting young women and causes ischemic changes in the vessel wall. It presents with complications of vessel lumen narrowing, such as ischemic pain, claudication, painful carotid, differences in systolic blood pressure (SBP) between arms, decreased brachial artery pulse, bruit over involved arteries, and stroke [1]. Signs and symptoms are not specific or sensitive for its diagnosis. Further testing is required in order to find the stroke etiology and tailor the management. In this case of TA presenting with an acute stroke, we describe a treatment-resistant patient, with short interval recurrences and the challenge of defining the stroke etiology as large vessel occlusive disease vs. arteritis flare.

## Case presentation

**Figure 1:**
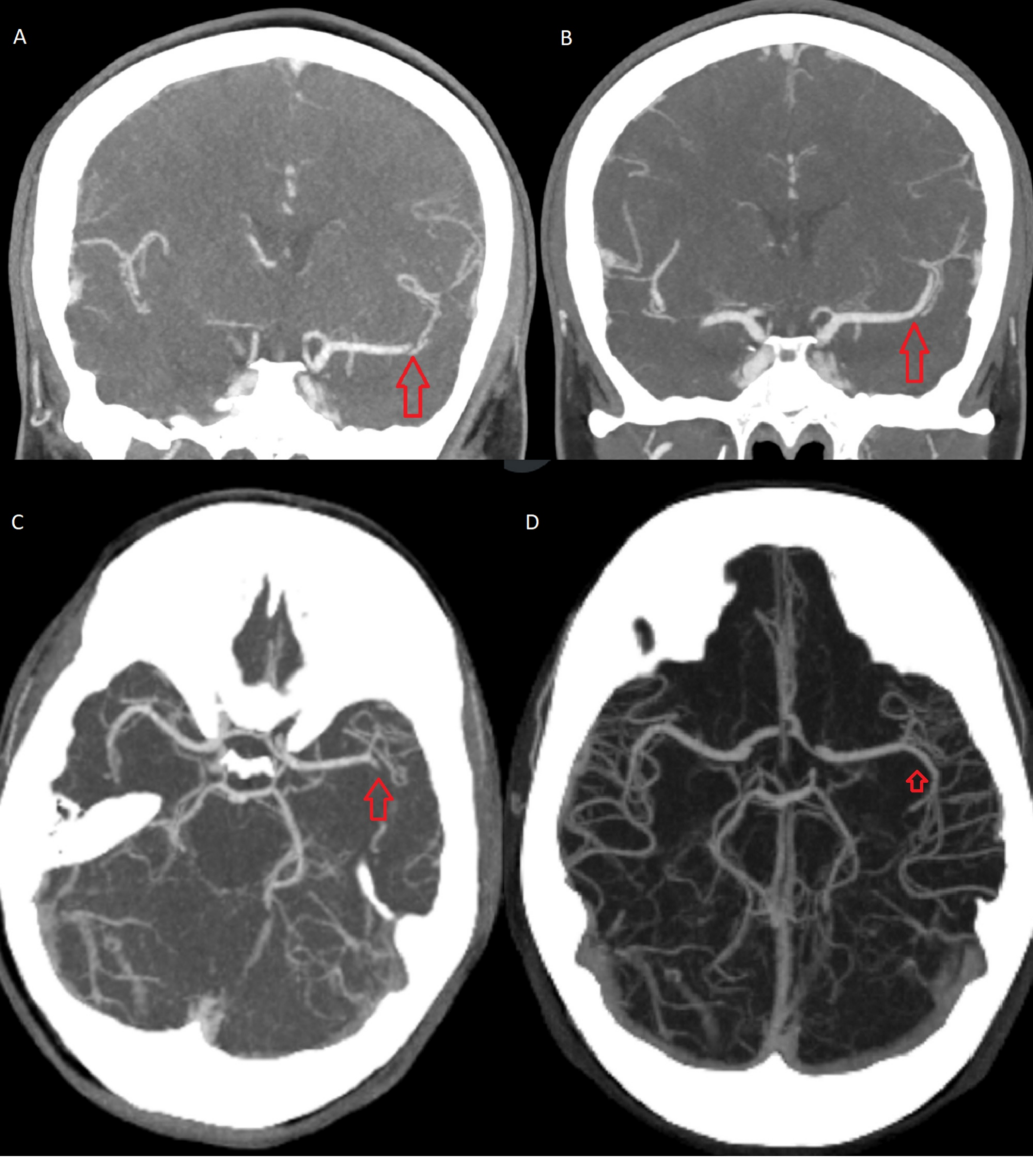
CT angiography of the brain vessels Left middle cerebral artery M2 branch thrombus (A) with maximum intensity projection (MIP) images (C) and resolution of the thrombus on follow-up imaging in three months (B) with MIP image (D).

**Figure 2:**
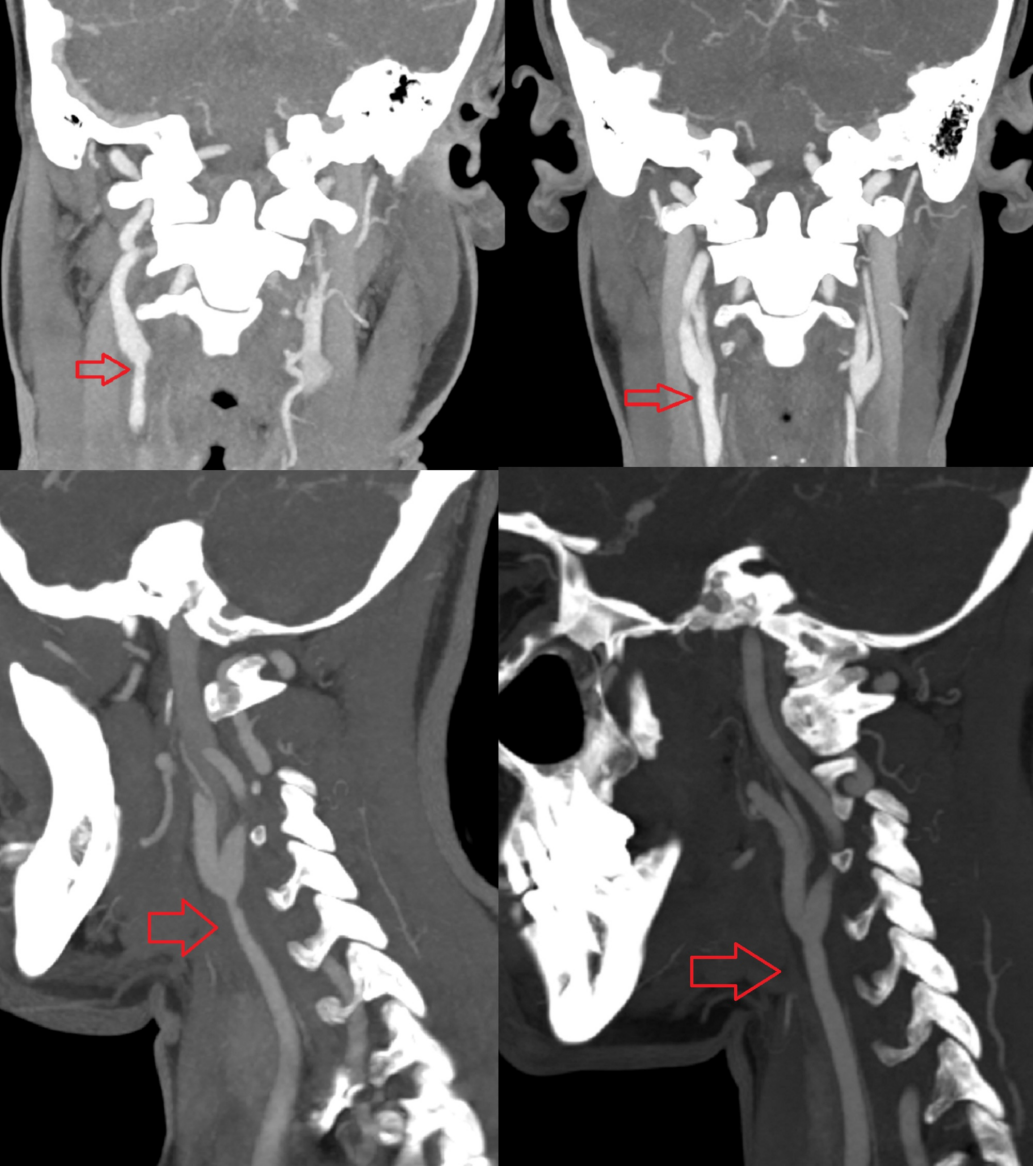
CT angiography of the neck vessels Right common carotid artery stenosis (right upper and maximum intensity projection [MIP] images on right lower) at the initial presentation and improvement of the stenosis after treatment on follow up images (left upper and MIP images on left lower) in three months.

**Figure 3:**
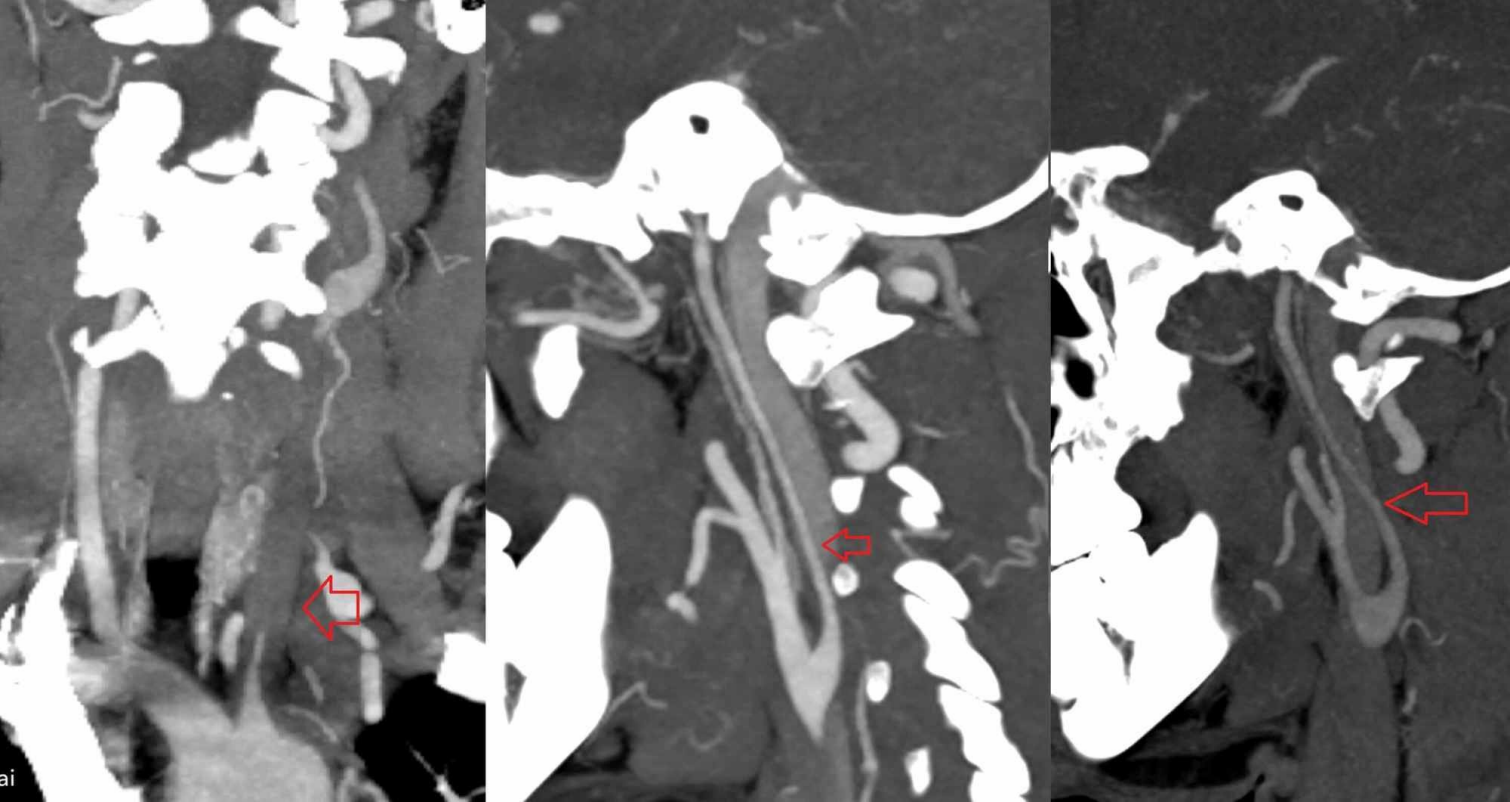
CT angiography of the left common carotid artery from aortic origin and left internal carotid artery Chronic occlusion of the left common carotid artery (right) and the chronic narrowing of the left internal carotid artery (middle and left).

A 25-year-old Caucasian woman with a history of TA admitted to our hospital with headache, agitation, and aphasia. She could not comprehend questions and write or repeat words. The patient was out of the window for tissue plasminogen activator (tPA) administration. The patient was diagnosed with TA at age 14 years. She had been on sarilumab and prednisone for four months before the presentation, and she was compliant with the treatment. Initial CT of the head did not reveal any acute abnormalities. CT angiography of the head and neck revealed occlusion of the M2 branch of the left middle cerebral artery (Figure 1), new since the prior study (two weeks prior to this presentation she had another stroke). The distal right common carotid artery was 70% stenotic (Figure 2), which was a progression from the prior study. The left common carotid artery (lCCA) was diffusely occluded and the left internal carotid artery was diffusely narrowed (Figure 3) but patent through its course which was deemed as chronic and stable when compared to the prior vessel imagings. 

**Figure 4:**
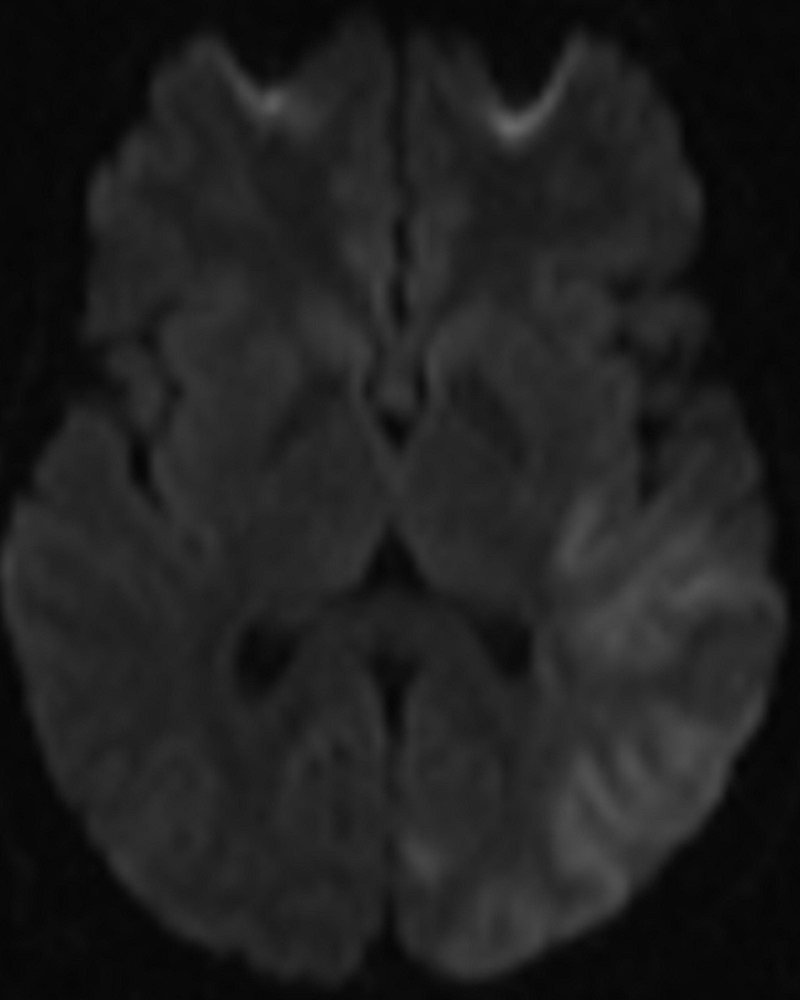
Diffusion MRI of the brain The MRI diffusion sequence shows hyperintensity of the left temporal lobe which is compatible with an acute stroke.

Given that the M2 thrombus was distal, the patient was not a candidate for mechanical endovascular treatment. MRI of the brain was remarkable for restricted diffusion within the gyri of the left temporoparietal lobes (Figure 4). Her initial erythrocyte sedimentation rate (ESR) was 17 (normal range [Nr]: 0-20 mm/hr) and C-reactive protein (CRP) was 1.46 (Nr<3.0 mg/L). These later increased to 28 and 13.25, respectively, on the second day of admission. The patient was started on 1,000 mg methylprednisolone intravenously for five days. Antiplatelet and high-dose statin treatments were also started. She had gradual improvement from being mute to having mild to moderate aphasia on day 3. 

**Figure 5:**
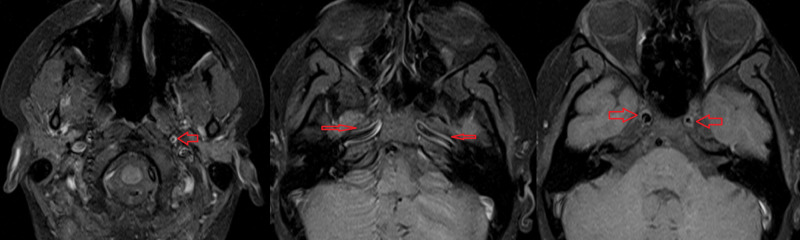
MRI with fat-saturated T1 post-contrast imaging of neck and brain arteries Contrast enhancement on the left distal common carotid artery (right). Active contrast enhancement on the wall of the petrous segment of bilateral internal carotid arteries (middle). Contrast enhancement on bilateral cavernous segments of internal carotid arteries (left).

At the end of the steroid treatment, the patient had an MRI brain of the head and neck with contrast, which showed active contrast enhancement on the wall of the left distal common carotid artery (Figure 5, right), the petrous segments (Figure 5, middle), and cavernous segments (Figure 5, left) of bilateral internal carotid arteries on fat-saturated T1 post-contrast imaging. These MRI brain findings confirmed the acute flare and failure of the disease-modifying anti-rheumatic drugs (DMARDs), sarilumab, due to the extensive large vessel wall inflammation. In the past, she failed mycophenolate mofetil, rituximab, tocilizumab, etanercept, adalimumab, methotrexate, and azathioprine. The patient was discharged on infliximab with a plan to have vascular imaging in three months. The follow-up imaging within three months was done with CTA, which showed persistent occlusion of the lCCA, but the right common carotid artery (rCCA) stenosis was resolved (Figure 2, left). Because of the given improvement of the stenosis, the patient did not have a follow-up MRI of the neck vessels to see the improvement of the vessel wall inflammation. 

## Discussion

Stroke is not rare (15.8%) and is a significant disability factor in the TA patient population [1]. Mechanisms of stroke in TA include large vessel occlusion/stenosis secondary to arterial thrombosis and vasculitis, carotid aneurysm/dissection, embolism from aortic regurgitation, hemodynamic origin due to vascular steal, vasospasm in hypertensive encephalopathy, distal carotid stump embolism, and moyamoya syndrome [1]. The diagnostic workup revealed the large vessel occlusion/stenosis secondary to arterial thrombosis and vasculitis as the stroke etiology of our patient. As can be seen, the etiological investigation process of the stroke can be challenging even after the diagnosis of TA. The management and secondary prevention of stroke is also difficult in TA. One of the main reasons for this is the limitations of markers reflecting disease activity, especially large artery wall inflammation, which is the primary pathological process of the disease [2]. If the etiology is the large vessel occlusion due to prior vessel injuries, then the secondary prevention should be antiplatelet treatment according to the stroke guidelines. However, if the etiology is active inflammation and the flare of the disease, the management may also include immunosuppressive treatment and reassessment of the DMARDs. In order to detect active disease process, acute-phase reactants (ESR and CRP) are practically used despite being shown to be neither sensitive nor specific enough for disease activity in TA [3].

Different non-invasive methods have been used to differentiate between acute disease processes and intra-arterial disease-related damage (atherosclerosis, stenosis, and occlusion). Ultrasonography (US), MRI, MR angiography, CT angiography (CTA), and positron emission tomography (PET) have been studied in patients with TA [4]. Current literature does not support one over another. Most of the time, the utilization of the tests relies on the preferences of clinicians, experiences with the modality, and the availability of the modality in the hospital setting. We have utilized MRI T1 with fat suppression of the head/neck with contrast to define the active inflammatory process. MRI helped us to elicit arterial wall thickness, the existence of edema, and contrast enhancement. The enhancement of the distal lCCA and bilateral common carotid arteries were significant for acute inflammation, which affected the DMARDs decision in our case. Even though some studies analyzed the utility of CTA, a large amount of radiation and ionized contrast will limit its utility. Another approach was taken by Herlin et al., who used the US to detect the active inflammation in the large artery wall. Contrast-enhanced US showed arterial wall enhancement of the lCCA in their case [2]. 18F-fluorodeoxyglucose-PET (FDG-PET) scanning is another studied modality. Even though initial studies showed promising results with PET, later studies could not confirm this, especially in regards to its sensitivity and specificity of stroke etiology in TA [3].

## Conclusions

Diagnosing the stroke etiology in TA patients, especially to identify whether the vessel wall pathology is an acute or chronic process causing an acute ischemic stroke, is an essential but challenging step. MRI T1 with fat suppression of the head/neck could be utilized to assess vessel caliber, patency, and for detection of inflammation within the vasculature, specifically in the vessel wall. 
